# On Some of the Initial Signs of Pleurisy
1Read at the Meeting of the Society, January 14th, 1903.


**Published:** 1903-03

**Authors:** F. H. Edgeworth

**Affiliations:** Assistant-Physician to the Bristol Royal Infirmary.


### ON SOME OF THE INITIAL SIGNS OF PLEURISY.[1]

BY

F. H. Edgeworth, M.B., B.A. (Cantab.), B.Sc. (Lond.),

*Assistant-Physician to the Bristol Royal Infirmary.*

I wish, in the following notes, to discuss some of the initial signs of pleurisy which are not described, or but barely

[1] Read at the Meeting of the Society, January 14th, 1903.

mentioned, in text books on medicine, though of considerable practical value and theoretical interest.

Hæmoptysis is occasionally one of the initial phenomena of a simple attack of pleurisy. With the onset of fever, pain in the side of the chest, and other symptoms, hæmoptysis may occur. Bright red, frothy blood may be expectorated, often accompanied by a little mucus. It is usually in small amount—a couple of drachms or so in the twenty-four hours. This hæmoptysis rarely lasts more than one day, though exceptionally it may go on in diminishing quantity for two or even three days.

The cause of this occasional hæmoptysis is a little doubtful. It is well known that a pleuritic rub and hæmoptysis may be among the first signs of pulmonary embolism; but in the cases I have mentioned an infarct was out of the question, for the heart was in each case normal, and there was no evidence of thrombosis in the general venous system. Next, a patch of pneumonia suggests itself as the explanation of the phenomena, but the clinical course of these cases is not that of a pneumonia; the blood expectorated is bright red, and it is an initial symptom only, whereas the rusty-coloured tenacious sputum of pneumonia is brought up on the second day, at earliest, of the disease. Pleurisy over a tubercular lesion of the lung is another possible explanation; but though an apical rub with hæmoptysis may be the only signs in the early stage of a case of phthisis, the cases described above are instances of basal pleurisy: the physical signs over the apices were normal, and the subsequent clinical course of the cases has not been that of a basal tubercular lesion.

It may be concluded, then, that pulmonary embolism, pneumonia, and an associated pulmonary tubercular lesion are not the causes of this transient hæmoptysis in pleurisy. It is probably due to hyperæmia and rupture of capillary vessels in the tissue of the lung immediately underneath the area of inflamed pleura.

The symptom is not of any bad significance; it speedily ceases, and it is of importance only in so far as, if the possibility of its occurrence is not known, it may lead one to unnecessarily suspect a graver lesion than is actually present.

The existence of pain in pleurisy is well enough recognised, but it is insufficiently appreciated that the pain often radiates along the nerves of the chest. Now, the lower six intercostal nerves pass into the upper two-thirds of the wall of the abdomen. It is therefore possible to entertain the opinion that peritonitis is present, and I have known cases where this was thought to be the case. This error is more easily made when the pleurisy is part of a pneumonia and the patient is seriously ill. The distinction, however, can be made by ascertaining that there is no tenderness of the skin of the abdomen or of the underlying peritoneum, and by finding the patch of pleurisy in the chest. On the other hand, if peritonitis as well as pleurisy be present, both peritoneum and pleura are tender on pressure.

The pain of pleurisy is not necessarily precisely over the pleuritic inflammation: all that it indicates is what intercostal nerves are implicated; and search must be made over the whole of the strip of pleura supplied by that nerve by palpating along the corresponding intercostal space, from the situation of the pain backwards towards the spine. An exact guide to the situation of the inflamed pleura is the area which is tender on pressure, and it is on listening there that a rub is heard. The skin itself is not tender; it may be pinched or pricked without the least pain, whilst the slightest pressure exerted on the underlying pleura is acutely painful. This tenderness on pressure often precedes the development of a pleuritic rub; thus there may be tenderness on pressure on the first day of the disease, and only on the succeeding one is a pleuritic rub audible. I think it may be said that a localised patch of tenderness in an intercostal space, which has suddenly developed, with pain, fever, and impaired costal movement, is very good evidence of commencing pleurisy. The tenderness does not last long, rarely more than two or three days, whether effusion follows or not. It is thus only an initial symptom.

I might say, in passing, that in the initial period of pericarditis there is similarly tenderness on pressure without any hyperæsthesia of the skin; but that in this case tenderness on pressure is not so surely evidence of inflammation of the serous membrane as in the case of the pleura, for it may be found where there is simply distension of the cardiac cavities; for instance, after severe exertion and in angina pectoris. The two chief affections, other than pleurisy, in which there is localised tenderness on pressure on the thoracic wall, are intercostal neuralgia and arthritis of the costo-vertebral joints. The former is a late phenomenon in neuralgia, and can hardly be mistaken for pleurisy, but the latter may be fairly acute. The tenderness, however, is quite close to the spinous processes, over the joints. Further, it is not accompanied by the other features of pleurisy. In the only two cases where I have seen it, the arthritis was of rheumatic origin, and readily cured by the administration of salicylates.

It may, perhaps, be added that the occurrence of pain and tenderness in pleurisy and pericarditis is fairly good clinical evidence that the intercostal nerves send sensory filaments to the parietal layer of the serous membranes, though as yet they have not been anatomically demonstrated.

There are many other phenomena occurring during an attack of pleurisy which might be discussed, but those to which I have referred are of special interest as liable to lead to errors in diagnosis or to be misinterpreted.

The discussion on this paper is reported on page 84.

